# Novel pyrazolothienopyridinones as potential GABA_A_ receptor modulators

**DOI:** 10.1007/s00706-023-03063-6

**Published:** 2023-04-13

**Authors:** Blanca Angelica Vega Alanis, Laurin Wimmer, Margot Ernst, Michael Schnürch, Marko D. Mihovilovic

**Affiliations:** 1 Institute of Applied Synthetic Chemistry, TU Wien, Getreidemarkt 9/163, 1060 Vienna, Austria; 2 Center for Brain Research, Medical University of Vienna, Spitalgasse 4, 1090 Vienna, Austria

**Keywords:** Drug research, Heterocycles, Scaffold Hopping, GABA_A_ Receptor Modulators, Electron-rich heterocyclic amines

## Abstract

The synthesis of novel pyrazolothienopyridinone derivatives as potential GABA_A_ receptor modulators was performed and is herein described. A crucial step of the synthesis involving handling unstable aminothiophenes was managed via two different synthetic strategies delivering a set of 8 target compounds.

**Graphical abstract:**

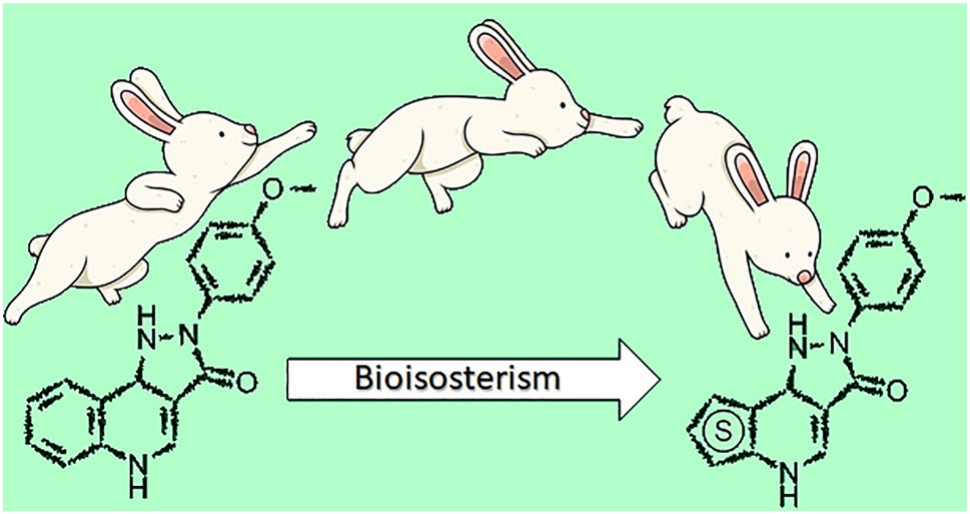

**Supplementary Information:**

The online version contains supplementary material available at 10.1007/s00706-023-03063-6.

## Introduction

**Fig. 1:**
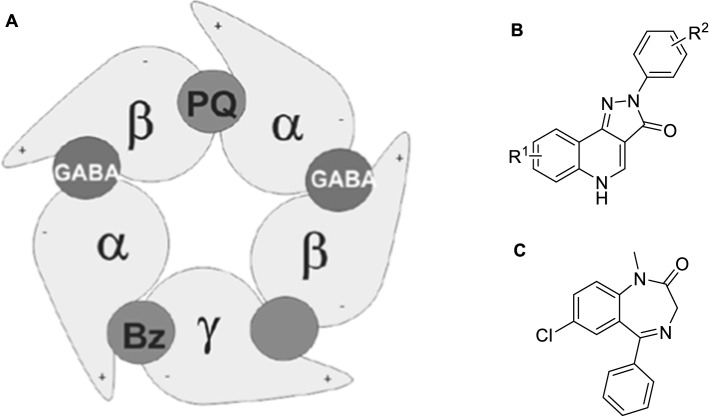
**A** Top view of the extracellular domain of a canonical GABA_A_ receptor. The binding sites for benzodiazepines (Bz) and Pyrazoloquinolinones (PQ) are explicitly shown. **B** general structure of PQs. **C** Diazepam as example for a benzodiazepin

GABA_A_ receptors are inhibitory chloride channels located in the mammalian central nervous system. They are targeted by many heavily used pharmaceuticals such as benzodiazepine-based sedatives, hypnotics, anxiolytics, barbiturate-based anticonvulsants, and general anesthetics of a broad range of chemotypes [1–3]. Structurally, these channels are pentamers, i.e. consisting of 5 subunits, which can be drawn from a pool of 19 different subtypes (α 1–6, β 1–3, γ 1–3, δ , ε , θ, π, and ρ). An example of a typical subunit arrangement is shown in Fig. 1.

Additionally, Fig. 1 shows the binding site of the endogenuous ligand GABA at the α-/β + binding site, besides alternative binding sites for benzodiazepines (Bz) and pyrazoloquinolinones (PQ). Besides those binding sites, additional possible binding sites have been identified which allow several ligand classes to interact with this family of receptors as well, making them highly promiscuous [1]. As a consequence, side effects are associated with all current therapies that target GABA_A_ receptors; thus, there is still the need to further investigate and develop improved ligands with subtype selectivity [4].

The complex structure of this receptor makes it a challenging task to selectively target one of the binding sites or even more to target a specific subunit subtype selectively [2, 5]. To elucidate this problem, PQs have been in the center of our attention for some time [1, 3, 5–9], and > 100 compounds were prepared with different substitution patterns on the A and D ring.

**Fig. 2:**
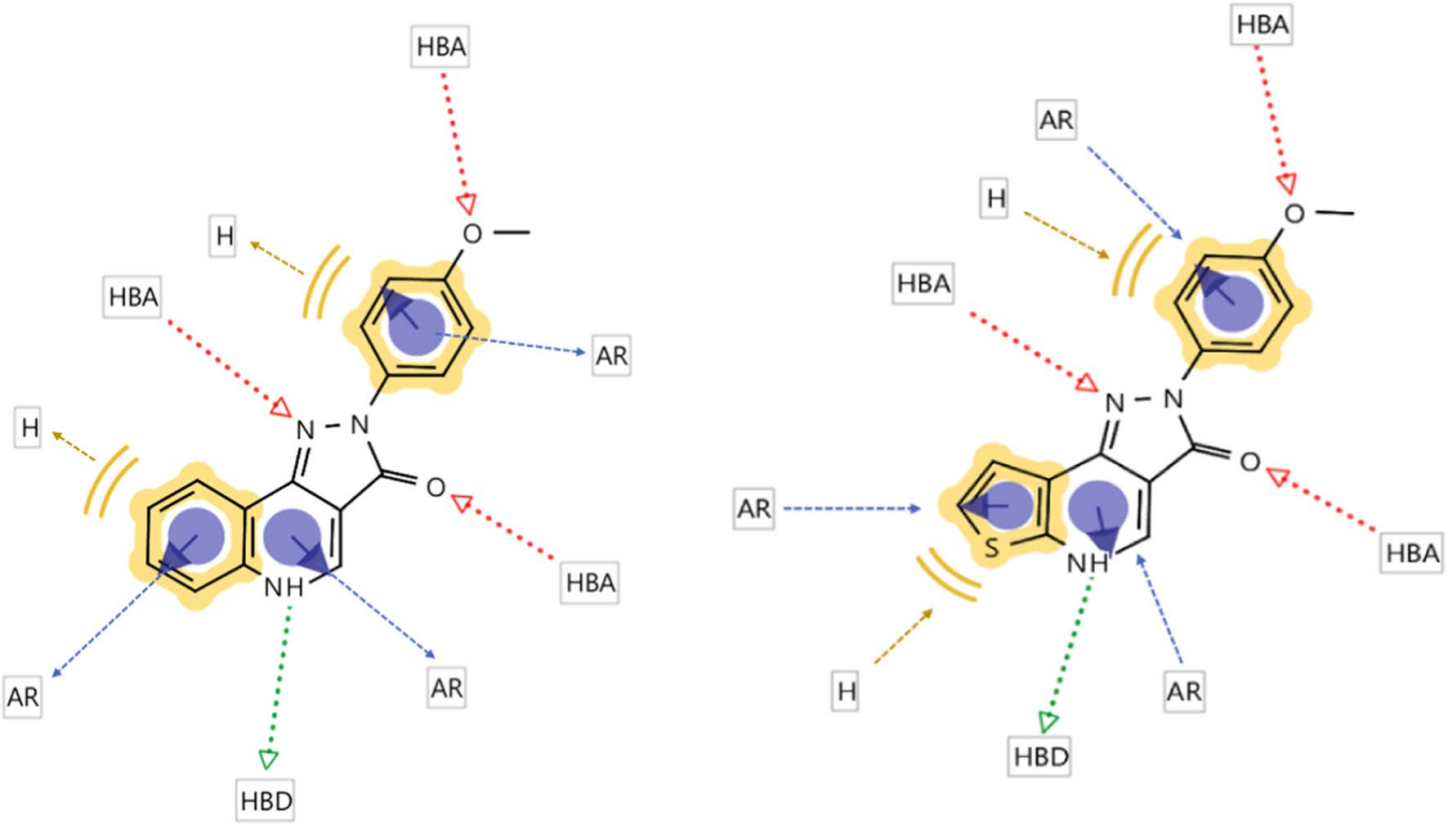
Putative pharmacophores of pyrazoloquinolinones and pyrazolothienopyridinones. *HBA* Hydrogen bond acceptor, *HBD* Hydrogen bond donor, *Ar* Aromatic ring, *H* Hydrophobic

In the present work, we targeted a more profound alteration of the scaffold, by exchanging the A ring with thiophene derivatives, since thiophene is a well-known bioisoster for the phenyl moiety [10]. The proposed derivatives would still maintain the same putative pharmacophores, as depicted in Fig. 2.

## Results and discussion

Traditionally, the initial step of a PQ synthesis starts from the accordingly substituted aniline** 1**, which is condensed with diethyl ethoxymethylene malonate (DEEMM,** 2**) to obtain a phenylaminomethylenemalonate intermediate **3** (Scheme 1) [11].

**Figure:**
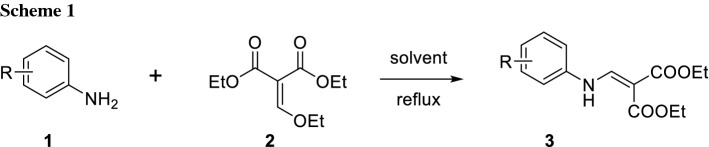

However, this route is not feasible for electron-rich heterocycles, such as thiophene, since some required amines of these heterocycles are unstable [12]. Therefore, several alternatives were explored to overcome this obstacle and obtain the thiophenyl aminomethylenemalonate intermediates (Scheme 2, compounds **6** and **9** and Scheme 3, compounds **15**, **16**, and **20**).

**Figure:**
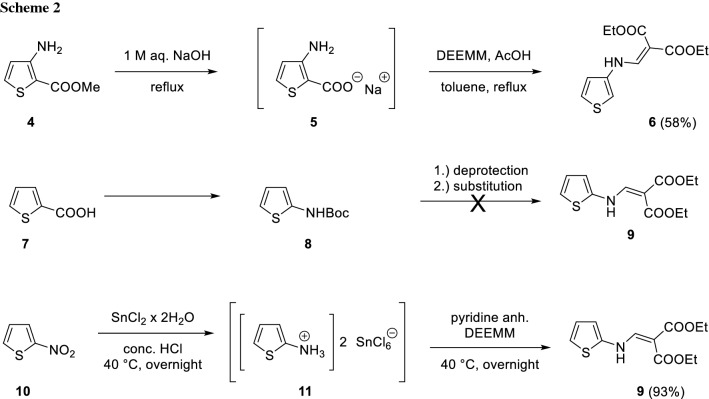

**Figure:**
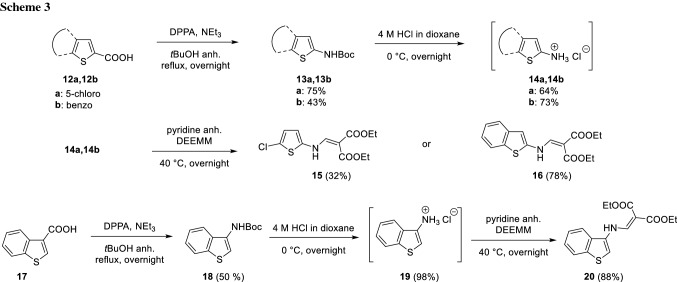

One of these compounds, namely **6**, was already reported in literature [5]. The synthesis started from methyl 3-aminothiophene-2-carboxylate (**4**), which was hydrolyzed to the corresponding carboxylic acid **5** and subsequently, in a one-pot fashion, decarboxylated and condensed to the desired intermediate** 6** at the same time (Scheme 2). This strategy is an attractive option, provided that such a starting material is obtainable, since three different transformations were done without the purification of each intermediate.

The regioisomeric product** 9** had to be prepared via an alternative route. Hence, it was tried to start from 2-thiophenecarboxylic acid (**7**) and synthesize Boc-protected 2-aminothiopene (**8**) via a Curtius rearrangement as reported in literature [13]. Deprotection and reaction with DEEMM would provide the desired intermediate **9**. Indeed, **8** could be obtained in 80% yield, however, deprotection attempts only led to a black precipitate which did not yield intermediate **9** after its treatment with dry pyridine and DEEMM. Consequently, a different method for the synthesis of intermediate **9** had to be chosen [12].

Reduction of 2-nitrothiophene** 10** with SnCl_2_‧2H_2_O was carried out and the resulting hexachlorostannate salt **11** was isolated in a crude form. Subsequently, pyridine was added to transform** 11** to 2-aminothiophene in situ followed by immediate condensation with DEEMM gave **9** in 93% yield.

For the remaining target compounds **15**, **16**, and **20** the strategy based on the Curtius rearrangement could be applied [14–17]. Substrates **12a**, **12b**, and **17** gave the Boc protected amines** 13a**,** 13b**, and** 18** in 91%, 43%, and 50% yield, respectively. Subsequently, the corresponding hydrochloride salts of the amines **14a**, **14b**, and **19** were obtained in 64%, 73%, and 98% yield via water-free deprotection with 4 M HCl in dioxane (Scheme 3). The isolation consisted of a simple filtration step and compounds **14a**, **14b**, and **19** were subjected immediately to the next step, where pyridine was used both as a solvent and as a base to regenerate the desired aminothiophene in situ. Anhydrous conditions were selected for this step since it was observed that the amine hydrochlorides were unstable when exposed to air. The target compounds **15**, **16**, and **20** were isolated in 32%, 78%, and 88% yield, respectively. Generally, the method is very convenient for handling potentially unstable aminothiophenes. The obtained aminothiophene hydrochlorides **14a**, **14b**, and **19** are easy to handle, and the use of high temperatures for the condensation with DEEMM is unnecessary, in contrast to other reported literature procedures [18]. It is noteworthy to mention that characterization of intermediates **14a**, **14b**, and **19** was not possible. ^1^H NMR of these salts did not show the expected ammonium signal and only noise was observed, most likely due to instability of the salts in deuterated solvents where traces of water or HCl (in case of CDCl_3_) are typically present.

Having compounds** 6**,** 9**,** 15**,** 16**, and** 20** in hand, the rest of the synthetic route was performed in a similar way to the classical synthesis of PQ derivatives [7] (Scheme 4). The aforementioned intermediates were thermally cyclized in Ph_2_O as solvent to give compounds** 21**,** 22**, and** 25–27**, which were then were chlorinated with phosphorous oxychloride, obtaining compounds **23**, **24**, and **28**–**30** (Scheme 4).

**Figure:**
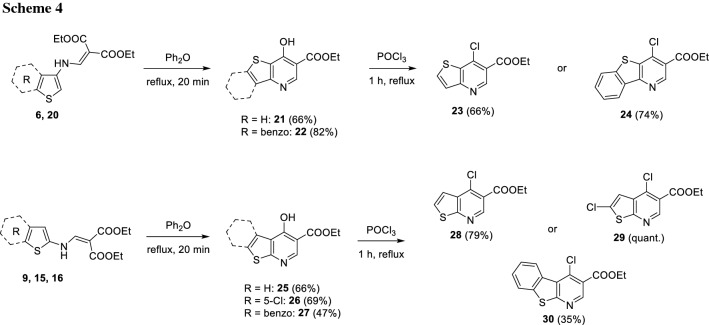

Afterwards, compounds **23**, **24**, and **28**–**30** underwent a nucleophilic aromatic substitution using 4-methoxyphenylhydrazine hydrochloride as the nucleophile adding NEt_3_ to generate the free hydrazine in refluxing EtOH overnight. Under these conditions, the hydrazine substituted compounds** 31**–**33** were obtained, however in low yields (Scheme 5). For further cyclization to the final products **34**–**36**, more forcing conditions had to be applied. These consisted of the stronger base potassium *tert*-butoxide and an increased temperature of 140 °C, which required DMAc as solvent (Scheme 5).

**Figure:**
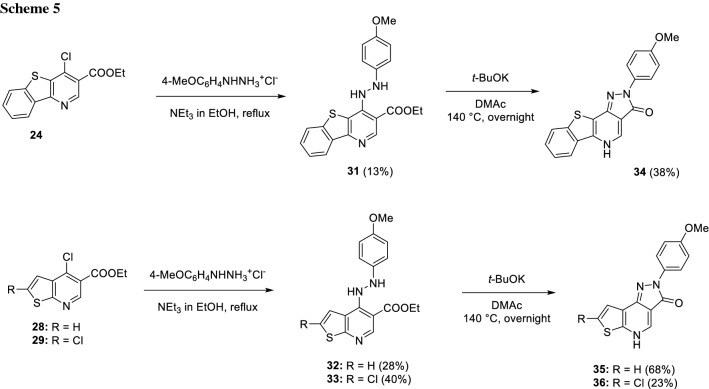

On the way to product **38**, the intermediate hydrazine** 37** was not isolated, but after nucleophilic substitution adding the stronger base potassium *tert*-butoxide to the NEt_3_ solution and increasing the temperature to 140 °C, which required DMAc as co-solvent, delivered** 38** in a one-pot fashion in 19% yield (Scheme 6) as previously reported in literature [5].

**Figure:**
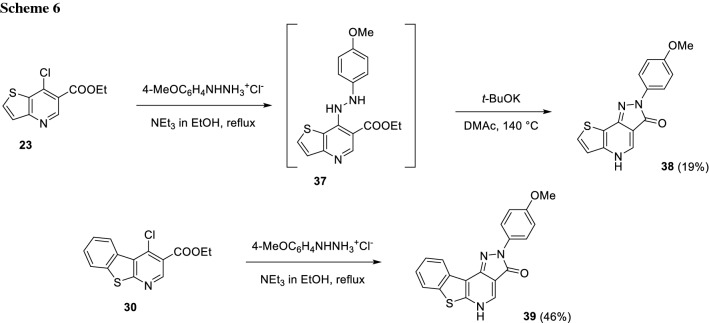

In the case of substrate** 30**, the hydrazine intermediate could not be detected, since even with NEt_3_ in ethanol, the second cyclization towards the final product occurred immediately delivering **39** in 46% yield (Scheme 6).

In conclusion, several strategies towards the synthesis of regioisomeric (in respect to the position of the sulfur) pyrazolothienopyridinones derivatives was achieved, which will further enable the development of new pyrazoloquinolinone derivatives. The major challenge, handling extremely electron rich thiophene amines was solved, which can serve as a blurprint for further derivatives with such features. The obtained target molecules will be subjected to biological testing and these results will be reported in due course.

## Experimental

All starting materials and reagents were purchased from commercial sources and used without further purification. Reactions were monitored by TLC on silica gel 60 F254 plates. Normal-phase column chromatography was performed on silica gel 60 (230 – 400 mesh). NMR spectra were recorded at 297 K in the solvent indicated, with 200, 400 and 600 MHz instruments, respectively, employing standard software provided by the manufacturer. ^1^H NMR and ^13^C NMR spectra were referenced to tetramethylsilane (TMS, *δ* = 0 ppm) by calibration with the residual organic solvent signals [19]. Accurate mass analysis (2 ppm mass accuracy) was carried out from 10 to 100 mg/dm^3^ solutions via LC-TOFMS measurements using an autosampler, an HPLC system with binary pumps, degasser, and column thermostat and ESI-TOF mass spectrometer. Melting points were determined with a Büchi Melting Point B-545 apparatus with a heating rate of 1 °C min^−1^ (70% onset point and 10% clear point) or on a Kofler Block apparatus. All melting points were obtained without additional recrystallization directly after flash column chromatography (FCC) with light petroleum (LP) and EtOAc and subsequent drying in high vacuum.

### General procedure 1: synthesis of heterocyclic *tert*-butyl-carbamate derivatives / Boc-protected heterocyclic amine derivatives

Heterocyclic *tert*-butyl-carbamate derivatives were synthesized according to a literature procedure [14]. In a previously dried 25 cm^3^ three-necked round-bottomed flask equipped with a magnetic stirring bar, reflux condenser, septa, and in an argon atmosphere, 1 eq. of the corresponding heterocyclic carboxylic acid was added, which was subsequently dissolved in dry *tert*-butanol. Afterward, 1.1 eq. of triethylamine was added, followed by the addition of 1.01 eq. of diphenylphosphoryl azide. The mixture was heated to reflux (85 °C) and was kept at refluxing temperature overnight. The initially light brown colored mixture became dark during the reaction time. After full conversion was observed on a TLC (DCM/MeOH 95:5 + 3 drops AcOH) using bromocresol green as a staining agent, the reaction mixture was poured into water, extracted 3 times with EtOAc and dried over anhydrous magnesium sulfate. The solvent was evaporated under reduced pressure, and the resulting product of this procedure was subjected to flash column chromatography using LP:CHCl_3_ = 60:40 as mobile phase to obtain the corresponding heterocyclic *tert*-butyl carbamates** 13a**,** 13b**, and** 18**.

### General procedure 2: synthesis of heterocyclic ammonium salts

Heterocyclic ammonium salts were synthesized according to a literature procedure [20, 21]. In a 10 cm^3^ dry reaction vial, equipped with a magnetic stirrer, septum, and an argon atmosphere, 1 eq. of the corresponding heterocyclic *tert*-butyl carbamate was added to a commercial solution of 4 N HCl in dioxane (13.8 eq.), and the mixture was stirred at room temperature until full consumption of the starting material was observed via TLC, using ninhydrin as a staining agent. Afterwards, a precipitate was observed in the reaction mixture. The reaction mixture was diluted with diethyl ether, and the product was collected by filtration, washed with diethyl ether, and dried in vacuo to afford the corresponding heterocyclic salts** 14a**,** 14b**, and** 19** as a product.

### General procedure 3: condensation with diethyl ethoxymethylenemalonate (DEEMM)

Diethylthiophenylamino methylene malonate derivatives were synthesized according to a literature procedure [14]. In a previously dried 30 cm^3^ reaction vial, equipped with a magnetic stirrer, septum, and an argon atmosphere, the corresponding heterocyclic salt was mixed with the required amount of diethyl ethoxymethylenemalonate, and afterwards, dry pyridine was added to the reaction vial. The reaction was heated at 40 °C and stirred overnight. After full conversion was observed via TLC using ninhydrin as a staining agent, water was added to the reaction mixture and extracted with chloroform. The organic phase was dried over magnesium or sodium sulfate and concentrated in vacuo, obtaining a crude material. The derivatives** 9**,** 15**,** 16**, and** 20** obtained via this procedure were purified via flash column chromatography using PE/EtOAc as mobile phase.

### General procedure 4: thermal cyclization

Ethylhydroxythienopyridine carboxylate derivatives were synthesized according to a literature procedure [3]. In a round-bottomed flask equipped with a magnetic stirrer and reflux condenser, the desired condensed substituted aniline derivate (1 eq.) was dispersed in approximately 2 cm^3^ of diphenyl ether and heated to 235 °C with a heating mantel. After full consumption was observed via TLC analysis using ninhydrin as a staining agent, the reaction mixture was adsorbed onto celite and purified via flash column chromatography, using mixtures of PE/EtOAc to yield the desired derivatives** 21**,** 22**, and** 25–27**.

### General procedure 5: synthesis of chlorothienopyridine carboxylate derivatives

Chlorothienopyridine carboxylate derivatives were synthesized according to a literature procedure [3]. The desired hydroxythienopyridine carboxylate was suspended in POCl_3_ and heated to reflux. After full conversion was observed via TLC analysis, the reaction mixture was cooled in an ice bath. Some pieces of ice were added slowly to the reaction mixture, followed by the careful addition of distilled water, ensuring the reaction temperature did not rise. Afterward, the pH of the reaction mixture was adjusted to 8 by adding either a saturated solution of Na_2_CO_3_ or solid Na_2_CO_3_. Then, the reaction mixture was extracted using EtOAc, dried over Mg_2_SO_4_, and concentrated in vacuo. The crude material obtained from this procedure was purified via flash column chromatography, using mixtures of PE/EtOAc to yield the purified halogenated derivatives** 23**,** 24**, and** 28–30**.

### General procedure 6: nucleophilic aromatic substitution of chlorothienopyridine carboxylates

Ethyl phenylhydrazineylthienopyridine carboxylate derivatives were synthesized according to a modified literature procedure [3]. In an 8 cm^3^ reaction vial equipped with a magnetic stirrer, 1.69 eq. of TEA, 4-methoxyphenylhydrazine hydrochloride (1.68 eq.), and 1 cm^3^ of dry EtOH were added and stirred for 2 min at room temperature. Afterward, 1 eq. of the desired chlorothienopyridine carboxylate was added, and the reaction mixture was heated to reflux and stirred overnight or until full consumption of the starting material was observed via TLC analysis. Afterward, distilled water was added to the reaction mixture, extracted 3 times with ethyl acetate, dried over magnesium sulfate, and concentrated in vacuo. The crude material was purified via flash column chromatography, using mixtures of PE/EtOAc as mobile phase to yield the desired derivatives** 31–33** and** 39**.

### General procedure 7: formation of pyrazolothienopyridinone derivatives

Pyrazolothienopyridinone derivatives were synthesized according to a literature procedure [22]. In a 50 cm^3^ flask with a septum, 1 eq. of the corresponding phenylhydrazineylthienopyridine carboxylate derivative and 1.5 eq. of *t*-BuOK were added and 3 times evacuated and flushed with argon. Afterward, 3 cm^3^ of the solvent were added to the flask. The reaction mixture was heated to 140 °C overnight. After full conversion was observed according to TLC analysis, the solvent was concentrated in vacuo. The product was purified via prep RP-HPLC. This procedure yielded derivatives** 34–36**.

#### Diethyl 2-[(thiophen-2-ylamino)methylene]malonate (9) [12, 17]

Compound** 9** was synthesized according to general procedure 3, using** 11** (1.82 g, 3.42 mmol) as starting material, 1 eq. of DEEMM (1.03 cm^3^, 3.42 mmol) and dry pyridine (20 cm^3^). After full consumption of the starting material was observed, water was added to the reaction mixture and in this particular reaction, a precipitate was formed during this step. The precipitate was filtered from the solvent and thoroughly washed with chloroform several times until obtaining a gray solid. Separately, the mother liquor was extracted with chloroform. Both chloroform phases were collected, dried over magnesium sulfate, and concentrated in vacuo. The crude product was purified via flash column chromatography, using a gradient of PE/EtOAc 95:5 to PE/EtOAc 90:10. Product **9** was obtained as yellow crystalline solid in 93% (0.86 g, 3.17 mmol) yield. M.p.: 40.3–40.5 °C (Ref. [12] 39–40 °C); *R*_*f*_ = 0.63 (PE/EtOAc = 9/1); HR-MS: *m/z* calcd. 270.0794 Da ([M + H]^+^), found 270.0795 Da, difference: 0.1 mDa = 0.35 ppm; ^1^H NMR (400 MHz, CDCl_3_): *δ* = 1.31 (t, *J* = 7.1 Hz, 3H, -CH_3_), 1.37 (t, *J* = 7.1 Hz, 3H, –CH_3_), 4.23 (q, *J* = 7.1 Hz, 2H, –CH_2_–), 4.30 (q, *J* = 7.1 Hz, 2H, –CH_2_–), 6.68 (ddd, *J* = 3.6, 1.5, 0.6 Hz, 1H, H5), 6.85–6.91 (m, 2H, H4 and H3), 8.24 (d, *J* = 13.2 Hz, 1H, NH–CH =), 11.11 (d, *J* = 13.2 Hz, 1H, –NH–) ppm; ^13^C NMR (101 MHz, CDCl_3_): *δ* = 14.4 (q, CH_3_), 14.5 (q, CH_3_), 60.3 (t, –CH_2_–), 60.7 (t, –CH_2_–), 94.0 (s, C_quat_), 114.5 (d, C5), 118.3 (d, C3), 126.7 (d, C4), 144.0 (s, C2), 154.4 (d, NH–CH =), 165.4 (s, C = O), 169.2 (s, C = O) ppm; Ref. [12] provides NMR data in DMSO-*d*_*6*_ as solvent.

#### Bis(2-thienylammonium)hexachlorostannate (11) [12]

Intermediate** 11** was synthesized according to a modified literature procedure. In a three-necked 50 cm3 round-bottomed flask, 2-nitrothiophene (**10**, 1.70 g, 13.2 mmol, 1 eq) was suspended in 20 cm^3^ of concentrated HCl and the mixture was heated to 50 °C and stirred until the substrate was dissolved. Afterwards, dihydrate stannous chloride (2 eq.) was added portionwise to the mixture, maintaining the reaction temperature between 40–45 °C by immersion in an ice bath. After the addition was complete, the cooling bath was removed, and the mixture was allowed to stir at 35 °C for 1.5 h. The reaction was monitored by TLC, showing full conversion at this point. The mixture was cooled overnight to 2 °C and** 11** was obtained as a gray precipitate in 26% yield (1.82 g, 3.43 mmol). Due to the instability of this compound, it was further converted immediately without full characterization.

#### *tert*-Butyl (5-chlorothiophen-2-yl)carbamate (13a) [23, 24]

Compound** 13a** was synthesized according to general procedure 1, using 5-chlorothiophene-2-carboxylic acid (**12a**, 1.0 g, 6.05 mmol) as starting material. **13a** was obtained as colorless crystalline solid in 75% (1.06 g, 4.54 mmol) yield. M.p.: 122.8–122.9 °C (Ref. [23] 142–143 °C); *R*_*f*_ = 0.38 (LP/CHCl_3_ = 6/4); ^1^H NMR spectral data is in accordance with literature data [24]; ^13^C NMR (101 MHz, CDCl_3_): *δ* = 28.0 (q, –(CH_3_)_3_), 80.4 (s, –C–CH_3_), 108.1 (d, C4), 117.9 (s, C5), 123.8 (d, C3), 139.4 (s, C2), 152.5 (s, C = O) ppm.

#### *tert*-Butyl 1-benzothiophen-2-ylcarbamate (13b) [15, 25]

Compound **13b** was synthesized according to general procedure 1, using** 12b** (1 g, 5.50 mmol) as starting material.** 13b** was obtained as colorless crystalline solid in 43% (0.59 g, 2.36 mmol) yield. M.p.: 101.7–102.2 °C (Ref. [25] 100–101 °C); *R*_*f*_ = 0.71 (LP/CHCl_3_ = 6/4); ^1^H NMR spectral data is in accordance with literature data [15]; ^13^C NMR (101 MHz, DMSO-*d*_*6*_): *δ* = 28.0 (q, –(CH_3_)_3_), 80.4 (s, –**C**–CH_3_), 104.5 (d, C3), 121.5 (d, C7), 121.7 (d, C4), 122.1 (d, C5), 124.3 (d, C6), 133.9 (s, C3a), 137.9 (s, C7a), 141.6 (s, C2), 152.4 (s, C = O) ppm.

#### 5-Chlorothiophen-2-aminium chloride (14a) [21]

Intermediate **14a** was synthesized according to general procedure 2,** 13a** (107 mg, 0.46 mmol) as starting material. Product** 14a** was obtained as brown pearly solid in 64% (50 mg, 0.30 mmol) yield. Due to the instability of this compound, it was further converted immediately without full characterization.

#### 1-Benzothiophen-2-aminium chloride (14b)

Intermediate **14b** was synthesized according to general procedure **2**, using **13b** (108 mg, 0.43 mmol) as starting material. The product was obtained as colorless pearly solid in 73% (58 mg, 0.32 mmol) yield. Due to the instability of this compound, it was further converted immediately without full characterization.

#### Diethyl 2-[[(5-chlorothiophen-2-yl)amino]methylene]malonate (15, C_12_H_14_ClNO_4_S)

Compound** 15** was synthesized according to general procedure 3, using **14a** (0.62 g, 3.66 mmol) as starting material, 1 eq. of DEEMM (1.1 cm^3^, 3.66 mmol) and dry pyridine (10 cm^3^). The product was purified via flash column chromatography, using PE/EtOAc 90:10 as mobile phase and obtained as yellow oil in 31% (0.35 g, 1.15 mmol) yield. *R*_*f*_ = 0.58 (PE/EtOAc = 85/15); LC–MS: *m/z* calcd. 304.76 ([M + H]^+^), found 304.10; ^1^H NMR (400 MHz, CDCl_3_): *δ* = 1.28 (t, *J* = 7.1 Hz, 3H, –CH_3_), 1.34 (t, *J* = 7.1 Hz, 3H, –CH_3_), 4.20 (q, *J* = 7.1 Hz, 2H, –CH_2_–), 4.26 (q, *J* = 7.1 Hz, 2H, –CH_2_–), 6.44 (d, *J* = 4.0 Hz, 1H, H3), 6.67 (d, *J* = 4.0 Hz, 1H, H4), 8.07 (d, *J* = 13.0 Hz, 1H, NH-CH =), 10.96 (d, *J* = 13.0 Hz, 1H, –NH–) ppm; ^13^C NMR (101 MHz, CDCl_3_): *δ* = 14.3 (q, CH_3_), 14.4 (q, CH_3_), 60.3 (t, –CH_2_–), 60.7 (t, –CH_2_–), 94.7 (s, C_quat_), 114.1 (d, C3), 123.1 (s, C5), 125.4 (d, C4), 141.4 (s, C2), 153.9 (d, NH–CH =), 165.0 (s, C = O), 169.0 (s, C = O) ppm.

#### Diethyl 2-[(1-Benzothiophen-2-ylamino)methylene]malonate (16, C_16_H_**17**_NO_4_S)

Compound **16** was synthesized according to general procedure 3, using** 14b** (0.17 g, 0.93 mmol) as starting material, 1 eq. of DEEMM (0.28 cm^3^, 0.93 mmol) and dry pyridine (5 cm^3^). The product was purified via flash column chromatography, using PE/EtOAc 90:10 as mobile phase. The product **16** was obtained as yellow crystalline solid in 77% (0.23 g, 0.72 mmol) yield. M.p.: 68.4–68.9 °C (Ref. [30] 69–70 °C); *R*_*f*_ = 0.39 (PE/EtOAc = 85/15); HR-MS: *m/z* calcd. 320.0951 Da ([M + H]^+^), found 320.0954 Da, difference: − 0.3 mDa = -1.06 ppm; ^1^H NMR (400 MHz, DMSO-*d*_6_): *δ* = 1.26 (t, *J* = 7.1 Hz, 6H, 2 -CH_3_), 4.18 (bs, 2H, 2 –CH_2_–), 7.22–7.30 (m, 2H, H3 and H6), 7.34 (t, *J* = 7.5 Hz, 1H, H5), 7.68 (d, *J* = 7.8 Hz, 1H, H4), 7.88 (d, *J* = 7.9 Hz, 1H, H7), 8.12 (s, 1H, NH-CH =), 11.06 (s, 1H, –NH-) ppm; ^13^C NMR (101 MHz, DMSO-*d*_6_): *δ* = 14.2 (q, 2 CH_3_), 59.8 (t, 2 –CH_2_–), 94.9 (s, C_quat_), 109.0 (d, C3), 122.3 (d, C7), 122.8 (d, C4), 123.7 (d, C6), 125.1 (d, C5), 133.5 (s, C3a), 138.8 (s, C7a), 143.9 (s, C2), 151.1 (d, NH–CH =), 164.7 (s, C = O), 166.46 (s, C = O) ppm; Both carbonyl signals were only observable on HMBC but did not appear in the ^13^C NMR. ^1^H NMR spectral data are in accordance with literature data [26].

#### 3-(*tert*-Butoxycarbonylamino)1-benzothiophen (18)

Compound** 18** was synthesized according to general grocedure 1, using** 17** (1 g, 5.51 mmol) as starting material.** 18** was obtained as yellow crystalline solid in 50% (0.68 g, 2.74 mmol) yield. M.p.: 104.6–105.2 °C (Ref [25] 104–105 °C); *R*_*f*_ = 0.31 (LP/CHCl_3_ = 6/4); ^1^H NMR (400 MHz, DMSO-*d*_*6*_): *δ* = 1.52 (s, 9H, –C– (CH_3_)_3_), 7.32–7.42 (m, 2H, H5, H7), 7.59 (s, 1H, H2), 7.91 (ddt, *J* = 7.5, 4.0, 2.0 Hz, 1H, H6), 8.09–8.16 (m, 1H, H4), 9.58 (s, 1H, –NH–) ppm; ^13^C NMR (101 MHz, DMSO-*d*_*6*_): *δ* = 28.1 (q, – (CH_3_)_3_), 79.5 (s, –C–CH_3_), 108.7 (d, C2), 121.3 (d, C4), 122.8 (d, C6), 123.7 (d, C7), 124.6 (d, C5), 130.5 (s, C3), 133.0 (s, C7a), 137.2 (s, C3a), 153.5 (s, C = O) ppm.

#### 1-Benzothiophen-3-aminium chloride (19)

Compound** 19** was synthesized according to general procedure 2, using** 18** (864 mg, 3.46 mmol) as starting material. Product** 19** was obtained as colorless pearly solid in 97% (625 mg, 3.39 mmol) yield. Due to the instability of this compound, it was further converted immediately without full characterization.

#### Diethyl 2-[(1-Benzothiophen-3-ylamino)methylene]malonate (20, C_16_H_17_NO_4_S) [25]

Compound** 20** was synthesized according to general procedure 3, using** 19** (0.59 g, 3.20 mmol) as starting material, 1 eq. of DEEMM (0.96 cm^3^, 0.93 mmol) and dry pyridine (10 cm^3^). The product was purified via flash column chromatography, using PE/EtOAc 90:10 as mobile phase and obtained as yellow crystalline solid in 88% (0.89 g, 2.81 mmol) yield. M.p.: 100.9–101.4 °C; *R*_*f*_ = 0.51 (PE/EtOAc = 85/15); HR-MS: *m/z* calcd. 320.0951 Da ([M + H]^+^), found 320.0959 Da, difference: − 0.8 mDa = − 2.55 ppm; ^1^H NMR (400 MHz, DMSO-*d*_6_): *δ* = 1.27 (m, 6H, 2 –CH_3_), 4.14 (q, *J* = 7.2 Hz, 2H, –CH_2_–), 4.25 (q, *J* = 7.1 Hz, 2H, –CH_2_–), 7.47 (ddd, *J* = 7.5, 1.4 Hz, 1H, H6), 7.52 ddd, *J* = 7.5, 1.4 Hz, 1H, H5), 7.73 (d, *J* = 7.4 Hz, 1H, H7), 7.78 (s, 1H, H2), 8.04 (d, *J* = 7.4 Hz, 1H, H4), 8.46 (d, *J* = 10.9 Hz, 1H, NH-CH =), 11.06 (d, *J* = 10.7 Hz, 1H, –NH–) ppm; ^13^C NMR (101 MHz, DMSO-*d*_6_): *δ* = 14.2 (q, 2 –CH_3_), 59.0 (t, –CH_2_–), 59.9 (t, –CH_2_–), 93.5 (s, C_quat_), 110.5 (d, C2), 119.7 (d, C7), 123.5 (d, C4), 124.8 (d, C5), 125.5 (d, C6), 132.0 (s, C3a), 132.4 (s, C3), 138.3 (s, C7a), 153.4 (d, NH–CH =), 164.7 (s, C = O), 167.8 (s, C = O) ppm.

#### Ethyl 7-hydroxythieno[3,2-*b*]pyridine-6-carboxylate (21) [26]

The title compound was prepared according to general procedure 4 starting using** 6** (3.7 g, 14.5 mmol, 1 eq.) as starting material. Compound** 21** was isolated via precipitation from the reaction mixture using LP (200 cm^3^). The precipitate was collected by filtration, washed with 2 × 50 cm^3^ of LP and dried in vacuo to give 1.57 g of** 21** (48%). The washing liquids were found to contain residual starting materials. After evaporation of volatiles the residue (Ph_2_O, substrate and traces of product) was heated again for one hour. After the work-up described above additional 560 mg (17%) of product could be isolated. Overall, 2.13 g (65%) brown solid of** 21** were obtained. M.p.: 242–245 °C (Ref. [17, 27] 259–262 °C); ^1^H NMR (400 MHz, DMSO-*d*_6_): *δ* = 1.26 (t, *J* = 7.1 Hz, 3H, CH_2_*CH*_*3*_), 4.20 (q, *J* = 7.1 Hz, 2H, *CH*_*2*_CH_3_), 7.29 (d, *J* = 5.4 Hz, 1H, ArH), 8.03 (d, *J* = 5.4 Hz, 1H, ArH), 8.49 (s, 1H, NCH), 12.78 (s, 1H, NH) ppm; ^13^C NMR (101 MHz, DMSO-*d*_6_): *δ* = 14.3 (q, CH_2_*CH*_*3*_), 59.7 (t, *CH*_*2*_CH_3_), 110.9 (s, C6), 118.8 (d), 130.7 (s), 133.6 (d), 143.1 (d, C5), 143.2 (s), 164.8 (s), 169.8 (COO) ppm.

#### Ethyl 4-hydroxybenzo[4,5]thieno[3,2-*b*]pyridine–3-carboxylate (22) [27]

Compound** 22** was synthesized according to general procedure 4, using** 20** (0.62 g, 1.94 mmol) and 2 cm^3^ of diphenyl ether. In this reaction, the purification via column chromatography was not performed since a precipitate appeared after adding PE to the reaction mixture. This precipitate was washed with LP and hexane several times and dried in vacuo*.* An analytical sample was recrystallized twice with DMSO, yielding** 22** as colorless solid in 82% (0.44 g, 1.59 mmol) yield. No further purification was required. M.p.: > 230 °C (Ref. [26] 255–257 °C); *R*_*f*_ = 0.38 (PE/EtOAc = 85/15); HR-MS: *m/z* calcd. 274.0532 Da ([M + H]^+^), found 274.0523 Da, difference: 1 mDa = 3.51 ppm; ^1^H NMR (600 MHz, DMSO-*d*_*6*_): *δ* = 1.30 (t, *J* = 7.1 Hz, 3H -CH_3_), 4.26 (q, *J* = 7.1 Hz, 2H, -CH_2_-), 7.61 (ddd, *J* = 7.5, 7.1, 1.2 Hz, 1H, H7), 7.64 (ddd, *J* = 8.1, 7.6, 1.4 Hz, 1H, H8), 8.14 (d, *J* = 7.9 Hz, 1H, H6), 8.41 (dd, *J* = 8.0, 1.6 Hz, 1H, H9), 8.55 (s, 1H, H2), 13.37 (s, 1H, -OH) ppm; ^13^C NMR (151 MHz, DMSO-*d*_*6*_): *δ* = 14.3 (q, –CH_3_), 60.1 (t, –CH_2_–), 112.9 (s, broad signal, C3), 122.7 (d, C9), 124.1 (d, C6), 125.4 (d, C7), 128.6 (d, C8), 130.3 (s, broad signal, C5a), 139.5 (s, C9a), 1430 (s, broad signal, C9b), 164.901 (s, C = O) ppm, C2 and C4 missing.

#### Ethyl 7-chlorothieno[3,2-*b*]pyridine-6-carboxylate (23) [26]

Compound** 23** was synthesized according to general procedure 5 using** 21** (2.45 g, 11 mmol) and 15 cm^3^ of POCl_3_. The crude product was purified via flash column chromatography, using a gradient of PE/EtOAc 90:10 delivering** 23** as brown solid in 66% (1.70 g, 7.0 mmol) yield. M.p.: 74–79 °C (Ref. [27] 82–83 °C); ^1^H NMR (200 MHz, CDCl_3_): *δ* = 1.44 (t, *J* = 7.1 Hz, 3H, CH_2_*CH*_*3*_), 4.46 (q, *J* = 7.1 Hz, 2H, *CH*_*2*_CH_3_), 7.60 (d, *J* = 5.5 Hz, 1H, ArH), 7.93 (d, *J* = 5.5 Hz, 1H, ArH), 9.14 (s, 1H, NCH) ppm; ^13^C NMR (50 MHz, CDCl_3_): *δ* = 14.4 (q, CH_2_*CH*_*3*_), 62.0 (t, *CH*_*2*_CH_3_), 120.0 (s, ArC), 125.9 (d, C2), 134.6 (s, ArC), 135.1 (d, C3), 139.2 (s, C7), 150.0 (d, C5), 158.3 (s, C8), 164.2 (s, COO) ppm. Spectral data were in agreement with those reported in the literature.

#### Ethyl 4-chlorobenzo[4,5]thieno[3,2-*b*]pyridine-3-carboxylate (24) [27]

Compound** 24** was synthesized according to general procedure 5 using** 22** (0.29 g, 1.12 mmol) and 1.5 cm^3^ of POCl_3_. The crude product was purified via flash column chromatography, using a gradient of PE/EtOAc 90:10 delivering** 24** as colorless crystalline solid in 74% (0.32 g, 1.10 mmol) yield. M.p.: 84–85 °C (Ref. [28] 88–89 °C); *R*_*f*_ = 0.37 (PE/EtOAc = 9/1); HR-MS: *m/z* calcd. 292.0194 Da ([M + H]^+^), found 292.0199 Da, difference: − 0.6 mDa = – 1.94 ppm; ^1^H NMR (400 MHz, CDCl_3_): *δ* = 1.47 (t, *J* = 7.1 Hz, 3H, –CH_3_), 4.50 (q, *J* = 7.1 Hz, 2H, –CH_2_-), 7.59 (ddd, *J* = 8.2, 7.1, 1.2 Hz, 1H, H8), 7.65 (ddd, *J* = 8.0, 7.2, 1.5 Hz, 1H, H7), 7.91 (dd, *J* = 8.0, 1.0 Hz, 1H, H6), 8.51 (dd, *J* = 7.9, 0.7 Hz, 1H, H9), 9.20 (s, 1H, H2) ppm; ^13^C NMR (101 MHz, CDCl_3_): *δ* = 14.4 (q, –CH_3_), 62.2 (t, –CH_2_–), 122.3 (s, C3), 123.2 (d, C6), 124.4 (d, C9), 125.8 (d, C8), 130.1 (d, C7), 134.6 (s, C9a), 135.5 (s, C4), 138.8 (s, C4a), 141.2 (s, C5a), 149.4 (d, C2), 154.9 (s, C9b), 164.2 (s, C = O) ppm.

#### Ethyl 4-hydroxythieno[2,3-*b*]pyridine-5-carboxylate (25) [29]

Compound** 25** was synthesized according to general procedure 4, using** 9** (0.75 g, 2.78 mmol) as starting material and 2 cm^3^ of diphenyl ether. Monitoring this reaction using ninhydrin as a TLC staining agent is a crucial step, since both the starting material and the resulting product have identical *R*_*f*_ values. The crude product was purified via flash column chromatography, using a gradient of PE/EtOAc 99:1 to PE/EtOAc 90:10 delivering** 25** as colorless solid in 66% (0.32 g, 1.83 mmol) yield. M.p.: 144.1–144.8 °C (Ref. [17] 152–153 °C); *R*_*f*_ = 0.63 (PE/EtOAc = 9/1); GC–MS: *m/z* = 223 (25, M^+^), 177 (100), 53 (45); HR-MS: *m/z* calcd. 224.0376 Da ([M + H]^+^), found 224.0378 Da, difference: − 0.2 mDa = − 0.74 ppm; ^1^H NMR (400 MHz, DMSO-*d*_*6*_): *δ* = 1.34 (t, *J* = 7.1 Hz, 3H, –CH_3_), 4.36 (q, *J* = 7.1 Hz, 2H, –CH_2_–), 7.48 (d, *J* = 5.9 Hz, 1H, H_2_), 7.68 (d, *J* = 5.9 Hz, 1H, H_3_), 8.70 (s, 1H, H6) ppm; ^13^C NMR (101 MHz, DMSO-*d*_*6*_): *δ* = 14.5 (q, –CH_3_), 61.6 (t, –CH_2_–), 108.2 (s, C5), 120.2 (d, C2), 125.5 (d, C3), 147.2 (d, C6), 161.9 (s, C7a), 164.4 (s, C3a), 167.9 (s, C = O) ppm, C4 missing. ^1^H NMR spectral data are in accordance with literature data [17]. C7a was only visible in HMBC, but not in ^13^C NMR.

#### Ethyl 2-chloro-4-hydroxythieno[2,3-*b*]pyridine-5-carboxylate (26, C_10_H_8_ClNO_3_S)

Compound** 26** was synthesized according to general procedure 4, using** 15** as substrate (1.14 g, 3.76 mmol) in 1 cm^3^ of diphenyl ether. The crude product was purified via flash column chromatography, using a gradient of PE/EtOAc 99:1 to PE/EtOAc 95:5 delivering** 26** as colorless solid in 69% (0.32 g, 0.68 mmol) yield. M.p.: 168–170 °C; *R*_*f*_ = 0.4 (DCM/MeOH 95/5); LC–MS: *m/z* calcd. 257.99 ([M + H]^+^), found 257.90; ^1^H NMR (400 MHz, CDCl_3_): *δ* = 1.46 (t, *J* = 7.1 Hz, 3H, -CH_3_), 4.49 (q, *J* = 7.1 Hz, 2H, –CH_2_-), 7.33 (s, 1H, H3), 8.85 (s, 1H, H6), 11.85 (s, 1H, –OH) ppm; ^13^C NMR (101 MHz, CDCl_3_): *δ* = 14.3 (q, –CH_3_), 62.3 (t, –CH_2_–), 106.0 (s, C5), 117.9 (d, C3), 122.7 (s, C2), 131.1 (s, C4), 148.4 (d, C6), 162.2 (s, C7a), 165.3 (s, C3a), 170.1 (s, C = O) ppm.

#### Ethyl 4-hydroxybenzo[4,5]thieno[2,3-*b*]pyridine-3-carboxylate (27, C_14_H_11_NO_3_S) [30]

Compound** 27** was synthesized according to general procedure 4, starting from** 16** (0.07 g, 0.19 mmol) and 2 cm^3^ of diphenyl ether. The starting material had to be dissolved in this case with the aid of the ultrasound bath. The crude product was purified via flash column chromatography, using a gradient of PE/EtOAc 99:1 to PE/EtOAc 95:5 delivering** 27** as beige solid in 47% (0.025 g, 0.09 mmol) yield. M.p.: 187.8–193.6 °C; *R*_*f*_ = 0.45 (PE/EtOAc 85/15); HR-MS: *m/z* calcd. 274.0532 Da ([M + H]^+^), found 274.0537 Da, difference: − 0.5 mDa = − 1.79 ppm; ^1^H NMR (400 MHz, CDCl_3_): *δ* = 1.49 (t, *J* = 7.1 Hz, 3H, –CH_3_), 4.52 (q, *J* = 7.1 Hz, 2H, –CH_2_–), 7.47–7.58 (m, 2H, H6 and H7), 7.76–8.00 (m, 1H, H8), 8.53–8.74 (m, 1H, H5), 8.98 (s, 1H, H2), 12.32 (s, 1H, –OH) ppm; ^13^C NMR (101 MHz, CDCl_3_): *δ* = 14.4 (q, –CH_3_), 62.3 (t, –CH_2_–), 106.3 (s, C3), 122.6 (s, C8), 125.7 (d, C6 or C7), 126.2 (d, C5), 127.2 (d, C6 or C7), 132.5 (s, C8a), 136.8 (s, C4b), 149.7 (d, C2), 164.4 (s, C9a), 167.2 (s, C4 or C4a), 167.5 (s, C4 or C4a), 170.4 (s, C = O) ppm.

#### Ethyl 4-chlorothieno[2,3-*b*]pyridine-5-carboxylate (28) [17]

Compound** 28** was synthesized according to general procedure 5, starting from** 25** (0.07 g, 0.31 mmol) and 1 cm^3^ of POCl_3_. For this product, the reaction time lasted 1 h and** 28** was obtained as yellow crystalline solid in 79% (0.06 g, 0.25 mmol) yield. M.p.: 68.0–68.5 °C (Ref. [17] 60–70 °C); *R*_*f*_ = 0.66 (PE/EtOAc = 85/15); GC–MS: *m/z* = 241 (35, M^+^), 196 (100), 133 (38); HR-MS: *m/z* calcd. 242.0037 Da ([M + H]^+^), found 242.0038 Da, difference: − 0.1 mDa = -0.58 ppm; ^1^H NMR (400 MHz, CDCl_3_): *δ* = 1.45 (t, *J* = 7.1 Hz, 3H, –CH_3_), 4.48 (q, *J* = 7.1 Hz, 2H, –CH_2_–), 7.55 (d, *J* = 6.1 Hz, 1H, H3), 7.66 (dd, *J* = 6.1, 0.4 Hz, 1H, H2), 9.01 (s, 1H, H6) ppm; ^13^C NMR (101 MHz, CDCl_3_): *δ* = 14.3 (q, -CH_3_), 62.0 (t, –CH_2_–), 120.9 (d, C3), 121.6 (s, C5), 129.1 (d, C2), 132.4 (s, C3a), 139.3 (s, C4), 148.2 (d, C6), 163.9 (s, C7a), 164.3 (s, C = O) ppm.

#### Ethyl 2,4-dichlorothieno[2,3-*b*]pyridine-5-carboxylate (29, C_10_H_7_Cl_2_NO_2_S)

Compound** 29** was synthesized according to general procedure 5, starting from** 26** (0.29 g, 1.12 mmol) and 2 cm^3^ of POCl_3_. The crude product was purified via flash column chromatography, using a gradient of PE/EtOAc 99:1 to PE/EtOAc 95:5 to give 29 as beige solid in quantitative yield (0.33 g, 1.12 mmol). M.p.: 63–65 °C; *R*_*f*_ = 0.66 (PE/EtOAc = 9/1); ^1^H NMR (600 MHz, CDCl_3_): *δ* = 1.44 (t, *J* = 7.1 Hz, 3H, -CH_3_), 4.46 (q, *J* = 7.1 Hz, 2H, –CH_2_–), 7.39 (s, 1H, H3), 8.94 (s, 1H, H6) ppm; ^13^C NMR (151 MHz, CDCl_3_): *δ* = 14.4 (q, –CH_3_), 62.2 (t, –CH_2_–), 119.8 (d, C3), 122.4 (s, C5), 132.4 (s, C2), 135.1 (s, C3a), 137.7 (s, C4), 148.5 (d, C6), 162.7 (s, C7a), 164.2 (s, C = O) ppm.

#### Ethyl 4-chlorobenzo[4,5]thieno[2,3-*b*]pyridine-3-carboxylate (30, C_14_H_10_ClNO_2_S)

Compound** 30** was synthesized according to general procedure 5, starting from** 27** (1.4689 g, 5.37 mmol) and 10 cm^3^ of POCl_3_. The crude product was purified via flash column chromatography, using a gradient of PE/EtOAc 95:5 delivering** 30** as yellow solid in 35% (0.55 g, 1.90 mmol) yield. M.p.: 70–72.3 °C; *R*_*f*_ = 0.45 (PE/EtOAc = 85/15); HR-MS: *m/z* calcd. 274.0532 Da ([M + H]^+^), found 274.0532 Da, difference: − 0.1 mDa = − 0.26 ppm; ^1^H NMR (400 MHz, DMSO-*d*_*6*_): *δ* = 1.39 (t, *J* = 7.1 Hz, 3H, –CH_3_), 4.42 (q, *J* = 7.1 Hz, 2H, –CH_2_–), 7.65 (ddd, *J* = 8.5, 7.2, 1.4 Hz, 1H, H6), 7.70 (ddd, *J* = 7.8, 7.5, 1.4 Hz, 1H, H7), 8.20 (dd, *J* = 7.8, 1.6 Hz, 1H, H8), 8.91 (dd, *J* = 7.9, 1.7 Hz, 1H, H5), 8.95 (s, 1H, H2) ppm; ^13^C NMR (101 MHz, DMSO-*d*_6_): *δ* = 14.0 (q, –CH_3_), 61.9 (t, –CH_2_–), 123.4 (s, C3), 123.6 (d, C8), 125.7 (s, C8a), 125.8 (d, C5), 126.0 (d, C6), 128.8 (d, C7), 131.4 (s, C4b), 137.4 (s, C4a), 138.0 (s, C4), 148.8 (d, C2), 163.8 (s, C = O), 164.7 (s, C9a) ppm.

#### Ethyl 4-[2-(4-methoxyphenyl)hydrazinyl]benzo[4,5]thieno[3,2-*b*]pyridine-3-carboxylate (31, C_21_H_19_N_3_O_3_S)

Compound** 31** was synthesized according to general procedure 6, starting from** 24** (0.063 g, 0.16 mmol). The crude product was purified via flash column chromatography, using a gradient of PE/EtOAc 95:5 to PE/EtOAc 85:15 and subsequently via RP-HPLC. Compound** 31** was obtained as orange solid in 13% (0.008 g, 0.021 mmol) yield. M.p.: 85–86 °C; *R*_*f*_ = 0.26 (PE/EtOAc = 9/1); LC–MS: *m/z* calcd. 394.12 ([M + H]^+^), found 394.05; HR-MS: *m/z* calcd. 394.1220 Da ([M + H]^+^), found 394.1214 Da, difference: 0.6 mDa = 1.6 ppm; ^1^H NMR (400 MHz, DMSO-*d*_*6*_): *δ* = 1.38 (t, *J* = 7.1 Hz, 3H, –CH_3_), 3.94 (s, 3H, –OCH_3_), 4.48 (q, *J* = 7.1 Hz, 2H, –CH_2_-), 7.26 (d, *J* = 9.0 Hz, 2H, H3’, H5’), 7.65 (ddd, *J* = 7.4, 0.8 Hz, 1H, H8), 7.72 (ddd, *J* = 7.5, 1.4 Hz, 1H, H7), 8.09 (d, *J* = 8.9 Hz, 2H, H2’, H6’), 8.16 (d, *J* = 7.9 Hz, 1H, H6), 8.50 (d, *J* = 7.7 Hz, 1H, H9), 9.18 (s, 1H, H2) ppm; ^13^C NMR (101 MHz, DMSO-*d*_6_): *δ* = 14.3 (q, –CH_3_), 56.0 (q, –OCH_3_), 61.9 (t, –CH_2_–), 115.3 (d, C3´, C5´), 119.4 (s, C3), 123.0 (d, C9), 123.6 (d, C6), 124.1 (s, C4a), 125.7 (d, C2´, C6´), 125.9 (d, C8), 129.8 (d, C7), 132.5 (s, C9b), 143.6 (s, C5a), 145.8 (s, C1´), 147.6 (d, C2), 148.4 (s, C4), 155.9 (s, C9a), 164.2 (s, C4´), 165.6 (s, C = O) ppm.

#### Ethyl 4-[2-(4-methoxyphenyl)hydrazinyl]thieno[2,3-*b*]pyridine-5-carboxylate (32, C_17_H_17_N_3_O_3_S)

Compound** 32** was synthesized according to general procedure 6, starting from** 28** (0.096 g, 0.40 mmol). The crude product was purified via flash column chromatography, using a gradient of PE/EtOAc 90:10 delivering** 32** as orange solid in 28% (0.038 g, 1.11 mmol) yield. M.p.: > 230 °C; *R*_*f*_ = 0.37 (PE/EtOAc = 85/15); LC–MS: *m/z* calcd. 342.09 ([M-H]^−^), found 342.17; HR-MS: *m/z* calcd. 342.0907 Da ([M]^+^), found 342.0904 Da, difference: + 0.3 mDa =  + 0.8 ppm; ^1^H NMR (400 MHz, DMSO-*d*_*6*_): *δ* = 1.15 (t, *J* = 7.1 Hz, 3H, -CH_3_), 3.92 (s, 3H, –OCH_3_), 4.25 (q, *J* = 7.1 Hz, 2H, –CH_2_–), 7.22 (d, *J* = 9.0 Hz, 2H, H3 ‘, H5 ‘), 7.63 (d, *J* = 6.1 Hz, 1H, H3), 8.00 (d, *J* = 9.0 Hz, 2H, H2 ‘, H6 ‘), 8.10 (d, *J* = 6.0 Hz, 1H, H2), 8.94 (s, 1H, H6) ppm; ^13^C NMR (101 MHz, DMSO-*d*_6_): *δ* = 14.0 (q, –CH_3_), 55.9 (q, –OCH_3_), 61.4 (t, –CH_2_–), 114.9 (d, C3’, C5’), 115.7 (s, C5), 120.0 (d, C3), 123.6 (s, C3a), 125.7 (d, C2´, C4´), 131.0 (d, C2), 146.5 (s, C1’), 147.4 (d, C6), 152.4 (s, C4), 163.5 (s, C4´), 165.2 (s, 7a), 165.5 (s, C = O) ppm.

#### Ethyl 2-chloro-4-[2-(4-methoxyphenyl)hydrazinyl]thieno[2,3-*b*]pyridine-5-carboxylate (33, C_17_H_16_ClN_3_O_3_S)

Compound** 33** was synthesized according to general procedure 6, starting from** 29** (0.058 g, 0.21 mmol). The crude product was purified via flash column chromatography, using PE/EtOAc 90:10 delivering** 33** as orange solid in 40% (0.032 g, 0.085 mmol) yield. M.p.: 126–127 °C; *R*_*f*_ = 0.47 (PE/EtOAc = 90/10); LC–MS: *m/z* calcd. 376.05 ([M + H]^+^), found 376.05; HR-MS: *m/z* calcd. 376.0517 Da ([M]^+^), found 376.0525 Da, difference: − 0.8 mDa = − 2.07 ppm; ^1^H NMR (400 MHz, CDCl_3_): *δ* = 1.25 (t, *J* = 7.1 Hz, 3H, –CH_3_), 3.93 (s, 3H, –OCH_3_), 4.33 (q, *J* = 7.1 Hz, 2H, –CH_2_–), 7.06 (d, *J* = 9.0 Hz, 2H, H3’, H5’), 7.40 (s, 1H, H3), 7.99 (d, *J* = 8.9 Hz, 2H, H2’, H6’), 8.97 (s, 1H, H6) ppm; ^13^C NMR (101 MHz, CDCl_3_): *δ* = 14.4 (q, –CH_3_), 55.9 (q, –OCH_3_), 61.9 (t, –CH_2_–), 114.7 (d, C3’, C5’), 117.2 (s, C5), 119.4 (d, C3), 123.8 (s, C3a), 126.0 (d, C2´, C4’), 147.2 (s, C1’), 148.2 (d, C6), 152.5 (s, C4), 163.9 (s, C4´), 164.6 (s, C7a), 165.9 (s, C = O) ppm.

#### 2-(4-Methoxyphenyl)-2,5-dihydro-3*H*-benzo[4,5]thieno[3,2-*b*]pyrazolo[3,4-*d*]pyridin-3-one (34, C_19_H_12_N_3_O_2_S)

Compound** 34** was synthesized according to general procedure 7, starting from** 31** (0.19 g, 0.48 mmol). Water was added to the reaction mixture and a precipitate appeared, which was filtered. From this step, 20.6 mg of the product were obtained. It was observed that some precipitate got trapped within the glass filter of the funnel, thus, the filter was washed with DMSO until the precipitate completely dissolved and the DMSO was evaporated with high vacuum. Additionally, the water phase was extracted with EtOAc, dried over magnesium sulfate, and concentrated in vacuo. The desired reaction product was also found on the organic phase and the residue collected from the funnel. The three collected crude fractions were purified via RP-HPLC delivering** 34** as yellow solid in 38% (0.064 g, 0.18 mmol) yield. M.p.: > 230 °C; *R*_*f*_ = 0.9 (DCM/MeOH = 9/1); LC–MS: *m/z* calcd. 348.08 ([M + H]^+^), found 348.15; HR-MS: *m/z* calcd. 348.0801 Da ([M + H]^+^), found 348.0809 Da, difference: 0.8 mDa = 2.29 ppm; ^1^H NMR (400 MHz, DMSO-*d*_*6*_): *δ* = 3.79 (s, 3H, –OCH_3_), 7.02 (d, *J* = 8.6 Hz, 2H, H3’, H5’), 7.62 (d, *J* = 8.1 Hz, 2H, H8, H7), 8.10 (d, *J* = 8.6 Hz, 2H, H2’, H4’), 8.19 (d, *J* = 9.0 Hz, 1H, H6), 8.48 (s, 1H, H9), 8.81 (s, 1H, H4), 13.74 (s, 1H, –NH–) ppm; ^13^C NMR (101 MHz, DMSO-*d*_6_): *δ* = 55.3 (q, –OCH_3_), 108.2 (s, C3a), 113.9 (d, C3’, C5’), 118.6 (s, C10a), 120.6 (d, C2’, C4’), 121.6 (d, C9), 124.0 (d, C6), 125.6 (d, C7), 127.6 (d, C8), 129.9 (s, C5b), 133.4 (s, C1’), 134.1 (s, C5a), 136.9 (s, C9a), 137.4 (d, C4), 141.8 (s, C10b), 156.1 (s, C4’), 160.5 (s, C = O) ppm; C5a only visible in HMBC.

#### 2-(4-Methoxyphenyl)-2,5-dihydro-3*H*-pyrazolo[3,4-d]thieno[2,3-*b*]pyridin-3-one (35, C_15_H_11_N_3_O_2_S)

Compound** 35** was synthesized according to general procedure 7, starting from** 32** (0.07 g, 0.31 mmol) and 1 cm^3^ of POCl_3_. In this case, the crude product was only washed with petroleum ether and no further purification was required. The crude product was purified via flash column chromatography, using a gradient of PE/EtOAc 99:1 to PE/EtOAc 95:5 delivering** 35** as red solid in 68% (0.009 g, 0.03 mmol) yield. M.p.: 181–182 °C; *R*_*f*_ = 0.3 (DCM/MeOH = 95/5); HR-MS: *m/z* calcd. 298.0645 Da ([M + H]^+^), found 298.0644 Da, difference: 0.1 mDa = 0.33 ppm; ^1^H NMR (400 MHz, DMSO-*d*_*6*_): *δ* = 3.91 (s, 3H, -OCH_3_), 7.21 (d, *J* = 8.9 Hz, 2H, H3’, H5’), 7.56 (d, *J* = 6.1 Hz, 1H, H8), 7.98 (d, *J* = 8.9 Hz, 2H, H2’, H6’), 8.06 (d, *J* = 6.1 Hz, 1H, H7), 8.95 (s, 1H, H4) ppm; ^13^C NMR (101 MHz, DMSO-*d*_6_): *δ* = 55.9 (q, –OCH_3_), 114.9 (d, C3’, C5’), 116.9 (s, C3a), 120.0 (d, C2’, C6’), 123.0 (s, C8a), 125.5 (d, C7), 130.8 (d, C8), 146.6 (s, C1´), 147.7 (d, C4), 152.8 (s, C8b), 163.4 (s, C4’), 165.0 (s, C5a), 166.6 (s, C = O) ppm.

#### 7-Chloro-2-(4-methoxyphenyl)-2,5-dihydro-3*H*-pyrazolo[3,4-*d*]thieno[2,3-*b*]pyridin-3-one (36, C_15_H_10_ClN_3_O_2_S)

Compound** 36** was synthesized according to general procedure 7, starting from** 33** (0.02 g, 0.51 mmol). The crude product was purified via RP-HPLC delivering** 36** as yellow solid in 23% (0.004 g, 0.012 mmol) yield. LC–MS: *m/z* calcd. 332.02 ([M + H]^+^), found 332.05; ^1^H NMR (600 MHz, DMSO-*d*_*6*_): *δ* = 3.77 (s, 3H, –OCH_3_), 6.98 (d, *J* = 8.6 Hz, 2H, H3’, H5’), 7.52 (s, 1H, H8), 8.07 (d, *J* = 8.5 Hz, 2H, H2’, H4’), 8.48 (s, 1H, H4) ppm; ^13^C NMR (151 MHz, DMSO-*d*_6_): *δ* = 55.3 (q, -OCH_3_), 108.3 (s, C3a), 113.7 (d, C3’, C5’), 114.9 (s, C3a), 118.9 (s, C8a), 119.3 (d, C8), 120.6 (d, C2’, C4’), 125.8 (s, C7), 133.9 (s, C1’), 141.8 (d, C4), 142.6 (s, C8b), 155.7 (s, C4’), 160.1 (s, C5a), 163.1 (s, C = O) ppm.

#### 2-(4-Methoxyphenyl)-2,5-dihydro-3*H*-benzo[4,5]thieno[2,3-*b*]pyrazolo[3,4-*d*]pyridin-3-one (39, C_19_H_13_N_3_O_2_S)

Compound** 39** was synthesized according to general procedure 6, starting from** 30** (62.7 mg, 0.18 mmol). In this case, after full consumption of the starting material, a precipitate was observed in the reaction mixture, which was filtered. NMR analysis confirmed that said precipitate corresponds to the pure reaction product as yellow powder in 46% (0.029 g, 0.083 mmol) yield. No further purification was performed. M.p.: > 300 °C; *R*_*f*_ = 0.4 (DCM/MeOH = 95/5); HR-MS: *m/z* calcd. 348.0801 Da ([M + H]^+^), found 348.0801 Da, difference: 0.0 mDa = 0.06 ppm; ^1^H NMR (400 MHz, DMSO-*d*_*6*_): *δ* = 3.80 (s, 3H, -OCH_3_), 7.05 (d, *J* = 8.8 Hz, 2H, H3’, H5’), 7.56 (t, *J* = 7.6 Hz, 1H, H8), 7.66 (t, *J* = 7.5 Hz, 1H, H9), 8.14 (d, *J* = 8.1 Hz, 1H, H7), 8.19 (d, *J* = 8.7 Hz, 2H, H2’, H4’), 8.57 (d, *J* = 7.9 Hz, 1H, H10), 8.80 (s, 1H, H4), 13.57 (s, 1H, –NH–) ppm; ^13^C NMR (101 MHz, DMSO-*d*_6_): *δ* = 55.3 (q, –OCH_3_), 110.4 (s, C3a), 113.9 (d, C3’, C5’), 114.8 (s, C10b), 120.5 (d, C2’, C4’), 123.0 (d, C6), 124.3 (d, C10), 125.9 (d, C9), 126.1 (d, C8), 132.4 (s, C6a), 133.4 (s, C1’), 134.5 (s, C10a), 137.3 (d, C4), 142.1 (s, C10c), 156.0 (s, C4’), 160.1 (s, C = O) ppm; C5a missing, C4 barely visible in ^13^C NMR but visible in HMBC.

### Supplementary Information

Below is the link to the electronic supplementary material.

Supplementary file1 (DOCX 15678 KB)

## Data Availability

NMR data of synthesized compounds are available in the supporting information.
