# Supplementary material for: Novel pyrazolothienopyridinones as potential GABAA receptor modulators
Source: Monatsh Chem. 2023 Apr 13;154(12):1427–39. doi: 10.1007/s00706-023-03063-6 (PMC10667146; doi:10.1007/s00706-023-03063-6)
Supplement: Supplementary file 1 — Supplementary file1 (DOCX 15678 KB) [file 706_2023_3063_MOESM1_ESM.docx]

**Supplementary Info**

**Novel** **pyrazolothienopyridinones as potential GABA_A_ receptor modulators**

**Blanca Angelica Vega Alanis^1^ ● Laurin Wimmer^1^ ● Margot Ernst^2^ ● Michael Schnürch*^,1^ ● Marko Mihovilovic^1^**

___

🖂 Michael Schnürch

michael.schnuerch@tuwien.ac.at

^1^ Institute of Applied Synthetic Chemistry, TU Wien, Getreidemarkt 9/163, 1060 Vienna, Austria

^2^ Center for Brain Research, Medical University of Vienna, Spitalgasse 4, A-1090 Vienna, Austria

**Table of content:**

1H and 13C spectra of synthesized compounds
